# Vascular Endothelial Glycocalyx Plays a Role in the Obesity Paradox According to Intravital Observation

**DOI:** 10.3389/fcvm.2021.727888

**Published:** 2021-11-02

**Authors:** Shingo Mitsuda, Kohji Uzawa, Marie Sawa, Tadao Ando, Takahiro Yoshikawa, Hideki Miyao, Tomoko Yorozu, Akira Ushiyama

**Affiliations:** ^1^Department of Anesthesiology, National Disaster Medical Center, Tokyo, Japan; ^2^Department of Anesthesiology, Kyorin University School of Medicine, Tokyo, Japan; ^3^Meiji Pharmaceutical University Graduate School of Pharmaceutical Sciences, Tokyo, Japan; ^4^Department of Anesthesiology, Saitama Medical Center, Saitama Medical University, Saitama-Ken, Japan; ^5^Department of Environmental Health, National Institute of Public Health, Saitama, Japan

**Keywords:** obesity paradox, sepsis, cranial window, microvascular circulation, glycocalyx layer

## Abstract

According to the “obesity paradox,” for severe conditions, individuals with obesity may be associated with a higher survival rate than those who are lean. However, the physiological basis underlying the mechanism of the obesity paradox remains unknown. We hypothesize that the glycocalyx in obese mice is thicker and more resistant to inflammatory stress than that in non-obese mice. In this study, we employed intravital microscopy to elucidate the differences in the vascular endothelial glycocalyx among three groups of mice fed diets with different fat concentrations. Male C57BL/6N mice were divided into three diet groups: low-fat (fat: 10% kcal), medium-fat (fat: 45% kcal), and high-fat (fat: 60% kcal) diet groups. Mice were fed the respective diet from 3 weeks of age, and a chronic cranial window was installed at 8 weeks of age. At 9 weeks of age, fluorescein isothiocyanate-labeled wheat germ agglutinin was injected to identify the glycocalyx layer, and brain pial microcirculation was observed within the cranial windows. We randomly selected arterioles of diameter 15–45 μm and captured images. The mean index of the endothelial glycocalyx was calculated using image analysis and defined as the glycocalyx index. The glycocalyx indexes of the high-fat and medium-fat diet groups were significantly higher than those of the low-fat diet group (*p* < 0.05). There was a stronger positive correlation between vessel diameter and glycocalyx indexes in the high-fat and medium-fat diet groups than in the low-fat diet group. The glycocalyx indexes of the non-sepsis model in the obese groups were higher than those in the control group for all vessel diameters, and the positive correlation was also stronger. These findings indicate that the index of the original glycocalyx may play an important role in the obesity paradox.

## Introduction

Obesity is a risk factor for the development of various diseases (1–6). The body mass index (BMI) is used to determine obesity; thus, BMI control is important from the perspective of lifestyle-related diseases. Studies have shown that obesity is associated with diabetes (1), impaired glucose tolerance (2), hypertension (3, 4), hypercholesterolemia, low high-density lipoprotein cholesterol (5), and the development and severity of ischemic heart disease (5, 6). However, in some serious conditions, the prognosis is better in patients with a higher BMI (7–11). In other words, obesity is not only a risk factor for the development of disease but also linked to a better prognosis in patients. This phenomenon is termed the obesity paradox.

The obesity paradox was first discussed with respect to the survival of patients undergoing dialysis in 1999 (7). Recent systematic reviews, meta-analyses, and a retrospective cohort study of a large patient database have revealed that a higher BMI appears to increase survival of patients with sepsis (8–10). Although clinical data on the obesity paradox have been reported for heart failure (8, 9), diabetes mellitus (10), and critical illness mortality (11), only a few experimental studies have been conducted, and the pathophysiological mechanism of the obesity paradox remains unknown. Therefore, research is required to understand the mechanisms that cause the obesity paradox.

In recent years, glycocalyx (GCX), a glycoprotein that covers the surface of vascular endothelial cells, has been a key topic of research in terms of the treatment of severe conditions because it plays an important role in maintaining the integrity of the vascular walls and preventing plasma leakage (12). Disruption of the GCX layer on endothelial cells occurs in a variety of serious conditions, such as ischemia-reperfusion, inflammation, sepsis, shock, hyper/hypovolemia, hyperglycemia, and surgery (12–14). Additionally, increased expression of disintegration markers in GCX may be associated with increased mortality in trauma patients (15). Furthermore, syndecan-1 (Sdc-1) and hyaluronic acid, which are important components of GCX, are released into the blood of patients with severe conditions due to increased hyperpermeability and low plasma colloid osmotic pressure (16, 17).

The destruction of the blood–brain barrier is related to the occurrence and deterioration of neurological dysfunction in ischemic stroke; it causes edema in the brain, despite the fact that the tight junction formed is normal, and has a negative effect on mortality. GCX plays an essential role in brain endothelial cell transport system and central nervous system in maintaining the integrity of the blood–brain barrier (18). Regarding the pathogenesis of obesity paradox, to gain a deeper understanding of the mechanism of obesity paradox, it may be useful to measure the degree of GCX destruction and the thickness of GCX in the brain.

In this study, we hypothesized that the GCX in obese mice is thicker and more resistant to inflammatory stress than that in non-obese mice. Therefore, we used intravital microscopy to elucidate the differences in vascular endothelial GCX among three groups of mice fed diets with different fat concentrations. We then clarified the *in vivo* functional and structural changes according to the degree of obesity of microvascular endothelial cells using intravital microscopy. The primary endpoint of this study was the presence of differences in the index of the GCX layer according to the BMI. The index is an aggregation of fluorescence-intensity signals from several GCX components, such as glycosaminoglycans and heparan sulfate. The secondary endpoints were changes in the index of the GCX layer and level of Sdc-1 (a GCX degradation marker) according to the degree of obesity under sepsis. We also quantified serum adiponectin, which is thought to exert anti-inflammatory effects in sepsis (19). Adiponectin is released exclusively from white adipose tissue (20) and is the most abundant adipose-specific adipokine (19).

## Methods

### Animal Obesity Model and Ethical Statement

Male C57BL/6N mice were purchased from Japan SLC, Inc. (Shizuoka, Japan). The mice were divided into three groups: low-fat (L, fat: 10% kcal), medium-fat (M, fat: 45% kcal), and high-fat (H, fat: 60% kcal) diet (D12450, D12451, D12492, Research Diets, Inc, New Brunswick, NJ) groups. The mice were fed the respective diet from 3 weeks of age, provided tap water acidified with hydrochloric acid *ad libitum*, and housed in individually ventilated cage systems (Super Mouse 1400TM Micro-Isolator Rack; Lab Products, Inc., Seaford, DE, USA) with a 12-h light/dark cycle. Thereafter, a chronic cranial window (CCW) was surgically installed at 8 weeks of age. The body fat percentage was measured in nine mice using veterinary computed tomography (CT) with built-in body fat measurement software (R_mCT2, Co, Rigaku, Tokyo, Japan). All experimental protocols were approved by the Committee for Animal Experiments at the National Institute of Public Health (protocol number 31-002) and were in accordance with the guidelines and laws for animal experiments in Japan.

### Chemicals and Reagents

Fluorescein isothiocyanate (FITC)-labeled wheat germ agglutinin (WGA) from *Triticum vulgaris* was purchased from Sigma-Aldrich Co. (St Louis, MO, USA). Ketamine hydrochloride, xylazine hydrochloride, and an adiponectin enzyme-linked immunosorbent assay (ELISA) kit were purchased from FUJIFILM Wako Pure Chemicals Industries, Ltd. (Osaka, Japan). The mouse-soluble CD138 (Sdc-1) ELISA kit was purchased from Diaclone SAS (Besançon, France).

### CCW Preparation

To visualize micro-vessels, a CCW was used, which is less sensitive to adipocytes than the dorsal skin chamber. To install the CCW, a hole of diameter 5 mm was drilled in the skull of mice, and a glass slide was placed on top of the CCW and fixed with resin (Supplementary Figure 1). During the surgical procedure, the mice were anesthetized via an intramuscular injection of a mixture of ketamine (90 mg/kg body weight) and xylazine (10 mg/kg body weight). The depth of anesthesia was assessed based on toe pinch responses. Under anesthesia, a cranial window of diameter 5 mm was made via durotomy and centered 2-mm posterior and 2-mm lateral from the bregma.

### Sepsis Model

A sepsis model was established by performing cecal ligation and puncture (CLP) on CCW mice obtained using the above method. The Appendix of the anesthetized mice was exposed. The apical 5-mm tip of the Appendix was ligated, and the tip was punctured with a 21G needle. The Appendix was replaced in the abdominal cavity, the peritoneum was sutured, and the abdomen was closed. The survival rate and body weight of the CLP-treated mice were recorded after 24 and 48 h.

### Measurement of Endothelial Glycocalyx Index

**Figure 1:**
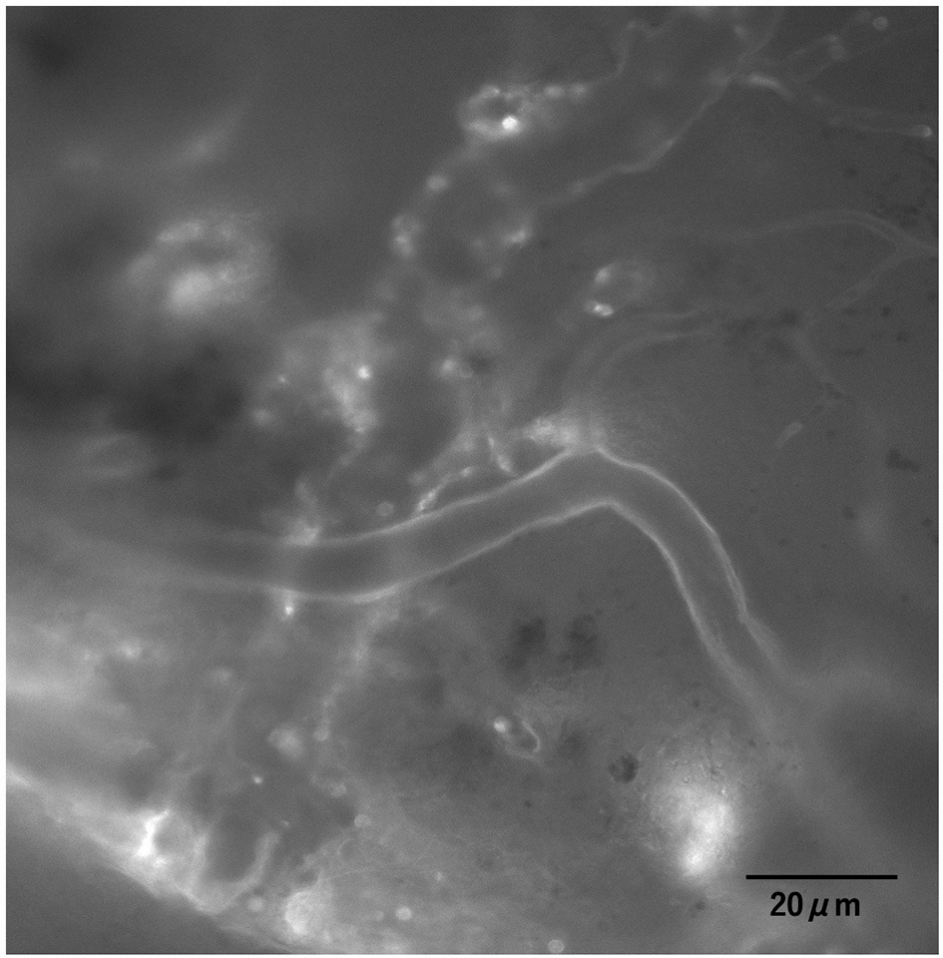
Images of the cerebral endothelial GCX. Cerebral endothelial GCX was illuminated with fluorescein isothiocyanate-labeled wheat germ agglutinin (FITC-WGA) lectin *in vivo* and observed through a cranial window using an intravital microscope.

At eight weeks of age, after inserting a CCW and performing CLP, the mice were left to stabilize for approximately 24 h and then injected with FITC-WGA via the tail vein. After 30 min, fluorescence images were obtained for each CCW (Figure 1). Vascular images within the CCW were observed using an all-in-one epifluorescence microscope (model BZ-9000; Keyence Co, Osaka, Japan) equipped with a high-sensitivity charge-coupled device camera and a ×20 long working distance objective lens (S PlanFL ELWD ADM 20×C, NA = 0.45; Nikon Co., Tokyo, Japan). Fluorescence images of WGA-FITC-stained regions were analyzed using ImageJ software (NIH, Bethesda, MD, USA). We defined the size of the FITC-WGA-positive layer as endothelial glycocalyx index (GCXI), which denotes the accumulation of several GCX components, such as glycosaminoglycans and heparan sulfate. The GCXI was measured as previously described (21), with minor modifications. Specifically, we selected arterioles (15–45-μm diameter) and measured the fluorescence intensity across three lines perpendicular to the artery walls in each CCW to compare the GCXI among the groups (Supplementary Figure 3A). Three lines perpendicular to the vessel wall were drawn and measured using ImageJ software. Because the obtained fluorescence-intensity values were not continuous data, a simple moving average of the fluorescence intensities at three points, including before and after, was calculated to smooth the fluorescence-intensity values at each of the obtained coordinates using an approximation formula. The inflection point was calculated based on the fitted curve, and the obtained data were differentiated twice. The two inflection points where the GCXI was measured were the inflection points in the range that matched the peak of the fluorescence curve on the original image and that were considered inflection points based on GCX staining. The two inflection points were subtracted from each other, and the value was used as the GCXI, which was considered the level of WGA-FITC accumulation in the endothelial GCX layer similar to that described in the measurement methods used in previous studies (22, 23). We defined the GCXI as the value multiplied by the length per pixel (Supplementary Figure 3B) and corrected by the actual distance (0.37744 μm/pixel). We calculated three independent GCXI values for individual mice and defined the mean values as the GCXI of the respective animals. To compare the measured GCXI with GCX thicknesses of various vessel diameters reported in previous studies, we also collected data on vessel diameter and GCX thickness from previous studies (21, 22, 24–32).

### Measurement of Sdc-1

After observation, blood samples were collected before and after CLP for biochemical tests. Blood was drawn from the buccal veins before CLP and from the heart after CLP. The blood concentration of Sdc-1 was quantified using a CD138 ELISA kit according to the manufacturer's instructions. Briefly, samples, standards, and diluted biotinylated anti-mouse CD138 antibody were added to precoated wells and incubated for 2 h at 25 °C. After three washes, horseradish peroxidase (HRP)-conjugated streptavidin was added, and the plate was incubated for 1 h at 25 °C. The substrate was then added, and the color was allowed to develop for 15–30 min. The absorbance at 450 nm was measured using a microplate reader (Bio-Rad Laboratories, Hercules, CA, USA). The concentration of Sdc-1 was calculated using a standard curve.

### Measurement of Adiponectin

Adiponectin samples were collected using the same method as that for Sdc-1. Adiponectin concentration in serum samples was determined using a commercially available adiponectin ELISA kit.

### Statistical Analysis

The measured data are expressed as mean ± standard deviation in figures and tables. The number of mice used in each experiment is described in the respective figure legends. Differences between survival rates were tested using the Kaplan–Meier analysis. The one-way analysis of variance followed by Tukey–Kramer multiple comparison test was used to compare the variables among the groups. Statistical significance was set at *p* < 0.05. Statistical tests were performed using JMP software package (JMP 14; SAS Inc., Cary, NC, USA). The age (weeks)/weight data of all mice are shown in Supplementary Table 1.

## Results

### Obesity Model

**Figure 2:**
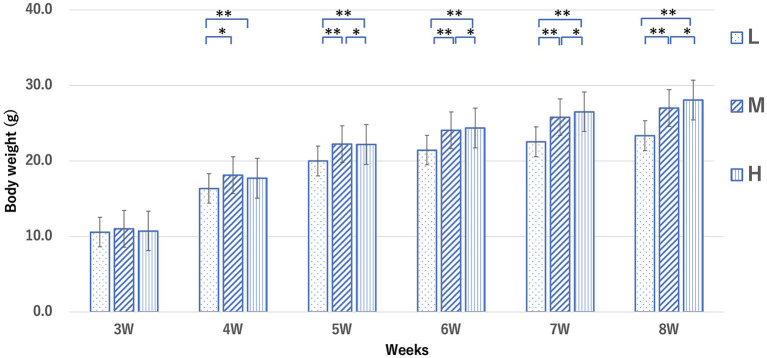
Weight changes in mice from each group from 3 to 8 weeks. Groups L, M, and H denote low-fat, medium-fat, and high-fat diet-fed mice, respectively. Data were analyzed using the Tukey–Kramer honestly significant difference test. *p < 0.05, **p < 0.01.

**Figure 3:**
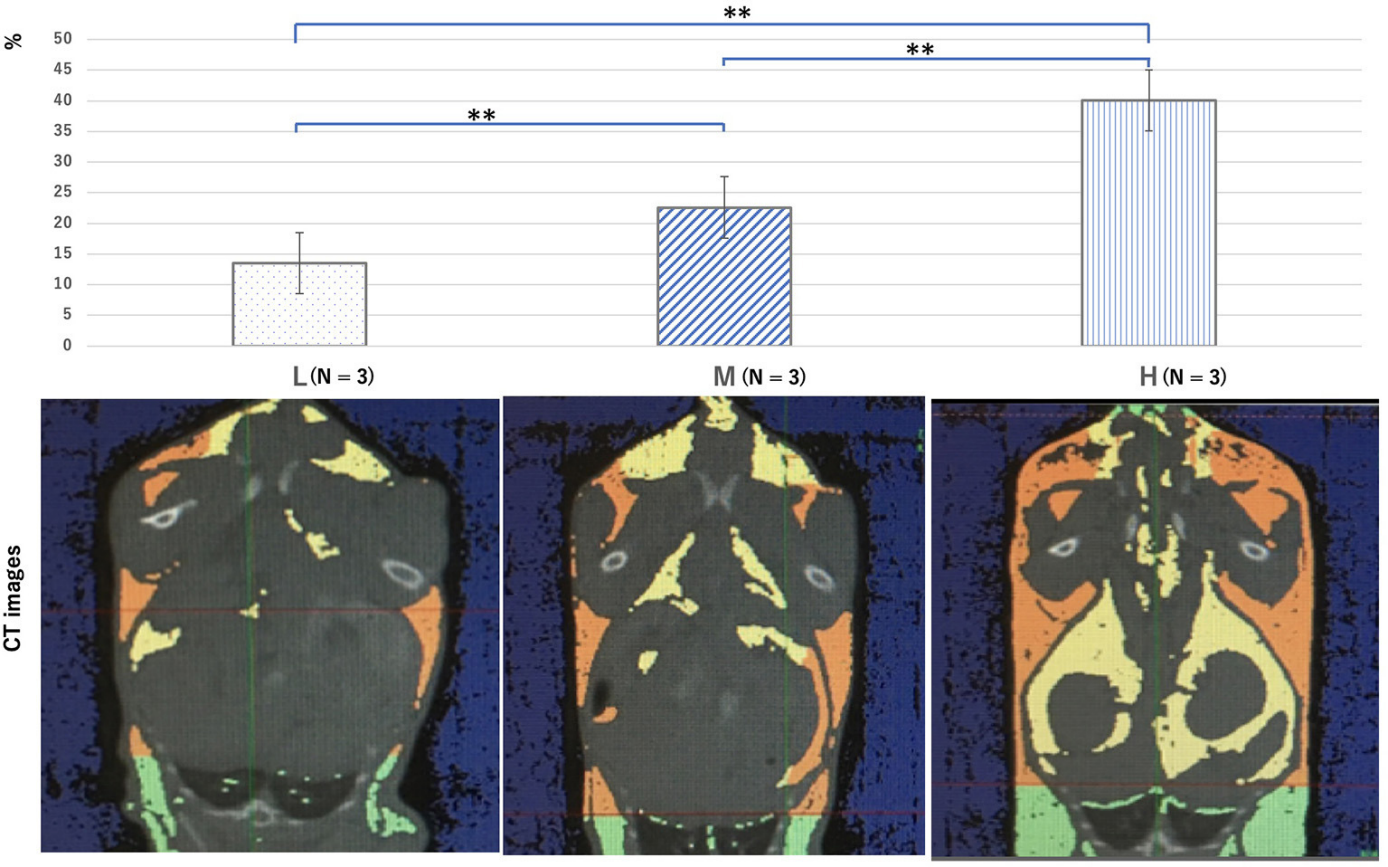
Computed tomography images showing a cross-section of the chest and abdomen, which were used to analyze the body fat percentage at 10 weeks for all groups. The yellow area shows visceral fat, the orange area shows subcutaneous fat, and the green area shows fatty tissue (not all were included in the fat percentage measured in this study). Groups L, M, and H denote low-fat, medium-fat, and high-fat diet-fed mice, respectively. Data were analyzed using Tukey–Kramer honestly significant difference test. **p < 0.01.

The mean weight of mice in the L, M, and H groups at 8 weeks was 21.8 ± 1.3, 24.8 ± 1.0, and 28.3 ± 1.8 g, respectively (Figure 2). After 6 weeks, the average weight in the M and H groups was significantly higher than that in the L group. The mean body fat percentage of each group, calculated by CT, was 13.5 ± 1.7%, 22.6 ± 4.1%, and 40.0 ± 1.2%, respectively (Figure 3). The body fat percentage of the H group was significantly higher than that of the M and L groups, and the body fat percentage of the M group was significantly higher than that of the L group. The mean blood pressure of the L, M, and H groups was 74.4 ± 7.16, 83.0 ± 8.32, and 81.8 ± 7.64 mmHg, respectively (Table 1). The total cholesterol level in the L, M, and H groups at 8 weeks was 96.1 ± 35.7, 162 ± 31.4, and 144 ± 24.2 mg/dl, respectively. The total cholesterol in the M and H groups was significantly higher than that in the L group. The results of blood counts and biochemical tests for each group are shown in Supplementary Table 2.

**Table 1:** Heart rate (HR), systolic blood pressure (sBP), mean blood pressure (mBP), and diastolic blood pressure (dBP) of mice in each group.

	**HR (bpm)**	**sBP (mmHg)**	**mBP (mmHg)**	**dBP (mmHg)**
L (N = 17)	666 ± 84.1	98.8 ± 6.37	74.4 ± 7.16	62.4 ± 9.27
M (N = 21)	715 ± 51.9	107 ± 9.90^*^	83.0 ± 8.32^**^	71.0 ± 9.07^*^
H (N = 15)	702 ± 10.7	105 ± 10.7	81.8 ± 7.64^*^	69.7 ± 7.53

*Data represent the mean ± standard error and were analyzed using Tukey–Kramer honestly significant difference test*.

* *p < 0.05*,

** *p < 0.01 vs. L. Groups L, M, and H denote low-fat, medium-fat, and high-fat diet-fed mice, respectively*.

### Index of Endothelial Glycocalyx

**Figure 4:**
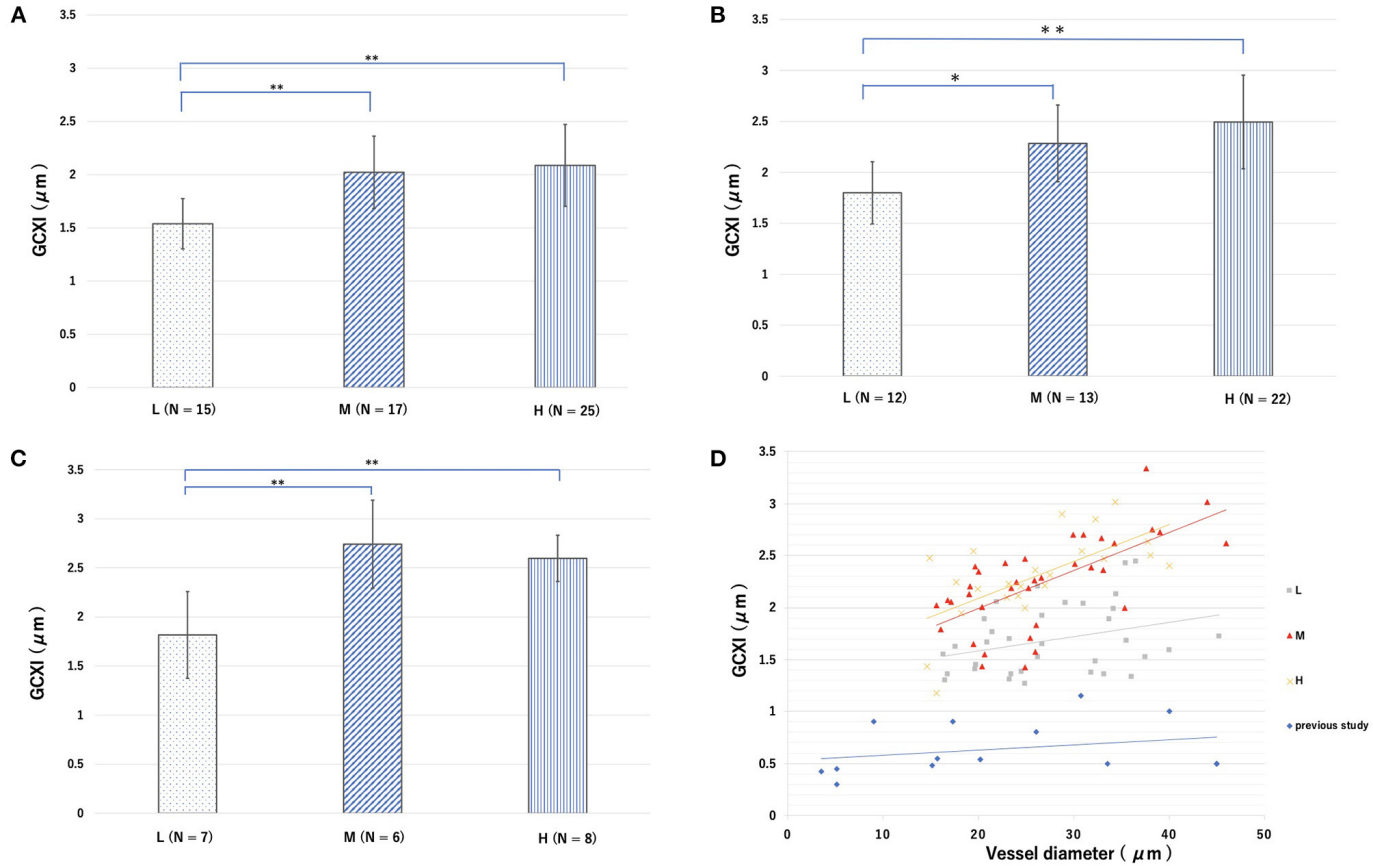
Groups L, M, and H denote low-fat, medium-fat, and high-fat diet-fed mice, respectively. Average endothelial glycocalyx index (GCXI) for vessel diameters of **(A)** <25 μm, **(B)** 25–35 μm, and **(C)** >35 μm in each group. Data were analyzed using Tukey–Kramer honestly significant difference test. *p < 0.05, **p < 0.01. **(D)** Relationship between the GCXI and blood vessel diameter in each group in this study and from previous studies. Gray, red, and yellow markers and lines indicate L, M, and H, respectively, and blue markers and lines indicate data from previous studies.

Under normal conditions, the cerebral endothelial GCX tagged with the FITC-WGA was imaged through a cranial window using a microscope (Figure 1). However, in cases of sepsis caused by CLP, clear images were difficult to obtain, and the GCXI was not available (Supplementary Figure 2B). The average GCXI for arterioles less than 25 μm in diameter in the L group was 1.53 ± 0.23 μm, whereas that in the M and H groups was 2.02 ± 0.34 and 2.09 ± 0.38 μm, respectively (Figure 4A). The average GCXI for arterioles measuring 25–35 μm in diameter in the L, M, and H groups was 1.79 ± 0.30, 2.28 ± 0.37, and 2.49 ± 0.45 μm, respectively (Figure 4B). The average GCXI for arterioles greater than 35 μm in diameter was 1.81 ± 0.44, 2.74 ± 0.44, and 2.59 ± 0.23 μm in the L, M, and H groups, respectively (Figure 4C). For all vessel diameters, the GCXI of the H and M groups was significantly higher than that of the L group (*p* < 0.05). Moreover, there was a positive correlation between vessel diameter and GCXI in the H (r^2^ = 0.42) and M groups (r^2^ = 0.40), but not in the L group (r^2^ = 0.098) (Figure 4D, Table 2 and Supplementary Table 5).

**Table 2:** Thickness of the glycocalyx (GCX) measured in various vessels in previous studies.

**Year**	**Author**	**Animal**	**Vessel**	**Method**	**Vessel diameter (μm)**	**GCX (μm)**
1996	Vink et al. (37)	Hamster	Muscle capillary	IM	5.1	0.3
2009	Potter et al. (25)	Mouse	Cremaster, mesenteric	μ-PIV	45.0	0.5
2011	Eno E Ebong et al. (26)	Hamster	Muscle capillary	RF/FS-TEM	5.1	0.5
2013	Lipowsky et al. (27)	Rat	Mesenteric	IM	33.5	0.5
2012	Wan-Y Yen et al. (28)	Rat	Mesenteric	IM	9.0	0.9
2013	Ivo Torres Filho et al. (29)	Rat	Cremaster	IM	15.1	0.47
2013	Michele D. Savery et al. (30)	Mouse	Cremaster	IM	20.2	0.5
2016	Ivo P. Torres Filho et al. (31)	Rats	Cremaster	IM	15.7	0.54
2017	Jin-Hui et al. (21)	Mouse	Cerebral penetrating A	TPLSM	30.7	1.2
2017	Jin-Hui et al. (21)	Mouse	Pial A	TPLSM	17.3	0.9
2017	Jin-Hui et al. (21)	Mouse	Capillary walls	TPLSM	3.5	0.4
2017	Kataoka et al. (22)	Mouse	Skin	IM	30.0	1.0
2018	Xiaoyuan et al. (32)	Mouse	Cremaster, mesenteric	IM	26.0	0.8

*IM, intravital microscopy; μ-PIV, microparticle image velocimetry; CLSM, confocal laser scanning microscopy; TPLSM, two-photon laser scanning microscopy; RF/FS-TEM, rapid freezing/freeze substitution transmission electron microscopy*.

### CLP Model

**Figure 5:**
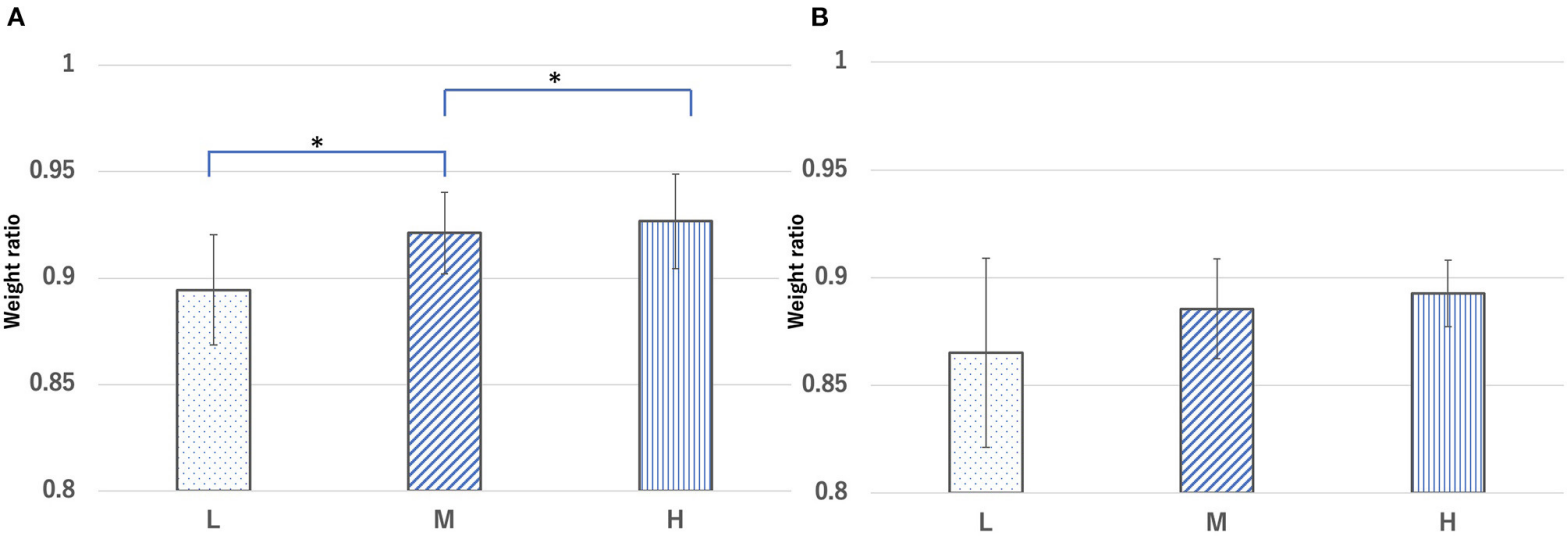
Ratio of body weight in each group [ratio = body weight **(A)** 24 h and **(B)** 48 h after cecal ligation and puncture (CLP) to body weight before CLP]. Groups L, M, and H denote low-fat, medium-fat, and high-fat diet-fed mice, respectively. Data were analyzed using Tukey–Kramer honestly significant difference test. *p < 0.05.

**Figure 6:**
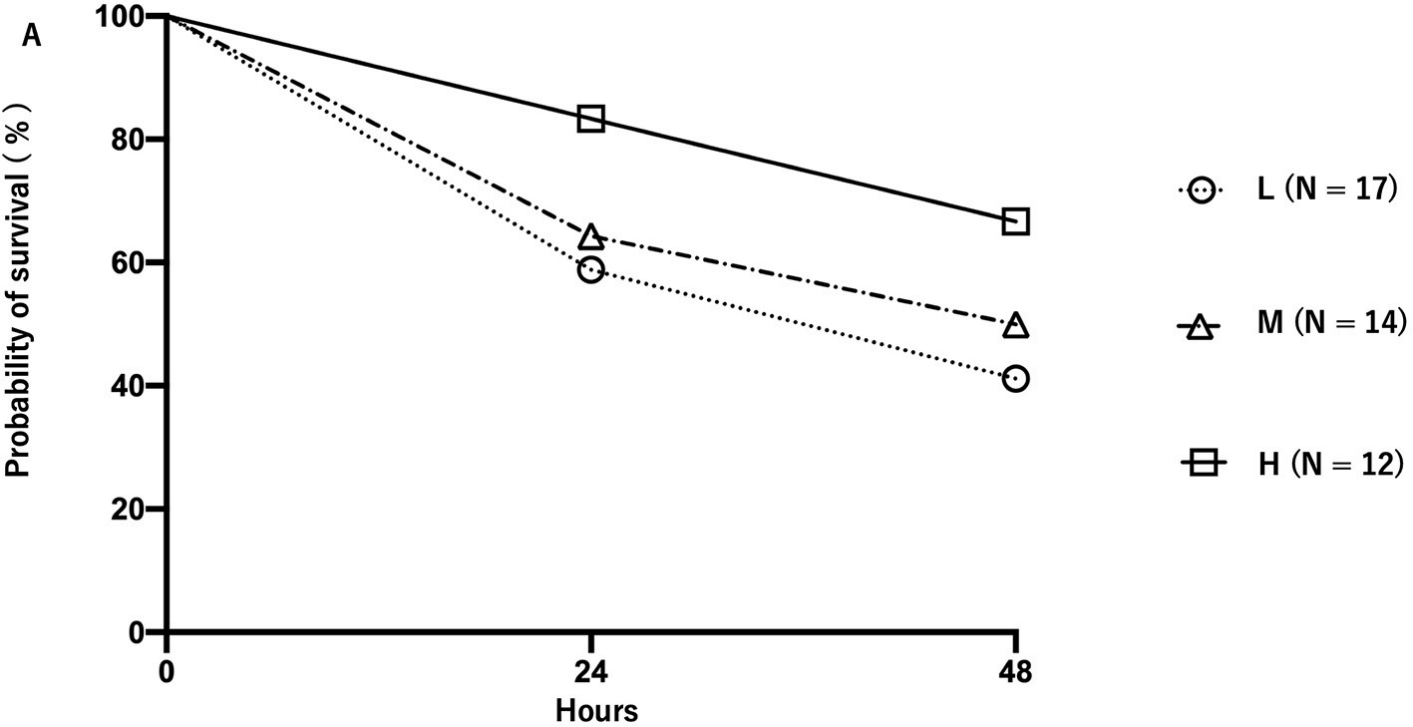
Survival rate of mice in each group after cecal ligation and puncture. Groups L, M, and H denote low-fat, medium-fat, and high-fat diet-fed mice, respectively. Circles, triangles, and squares represent the L, M, and H groups.

The ratio of body weight at 24 h after CLP to that before CLP was 0.89 ± 0.026, 0.92 ± 0.019, and 0.92 ± 0.022 for the L, M, and H groups, respectively. The body weight ratio of the H and M groups 24 h after CLP was significantly higher than that of the L group (*p* < 0.05) (Figure 5A). The ratio of body weight at 48 h after CLP to that before CLP was 0.86 ± 0.043, 0.88 ± 0.023, and 0.89 ± 0.015 for the L, M, and H groups, respectively (Figure 5B). The body weight of mice in each group before and after CLP is shown in Supplementary Table 3. The survival rate of mice at 24 h after CLP was 58.8, 64.2, and 83.0% for the L, M, and H groups, respectively, whereas the survival rate at 48 h was 41.1, 50.0, and 66.6%, respectively (Figure 6). There was no significant difference in the survival rate (*p* = 0.17) among the three groups.

### Adiponectin

**Figure 7:**
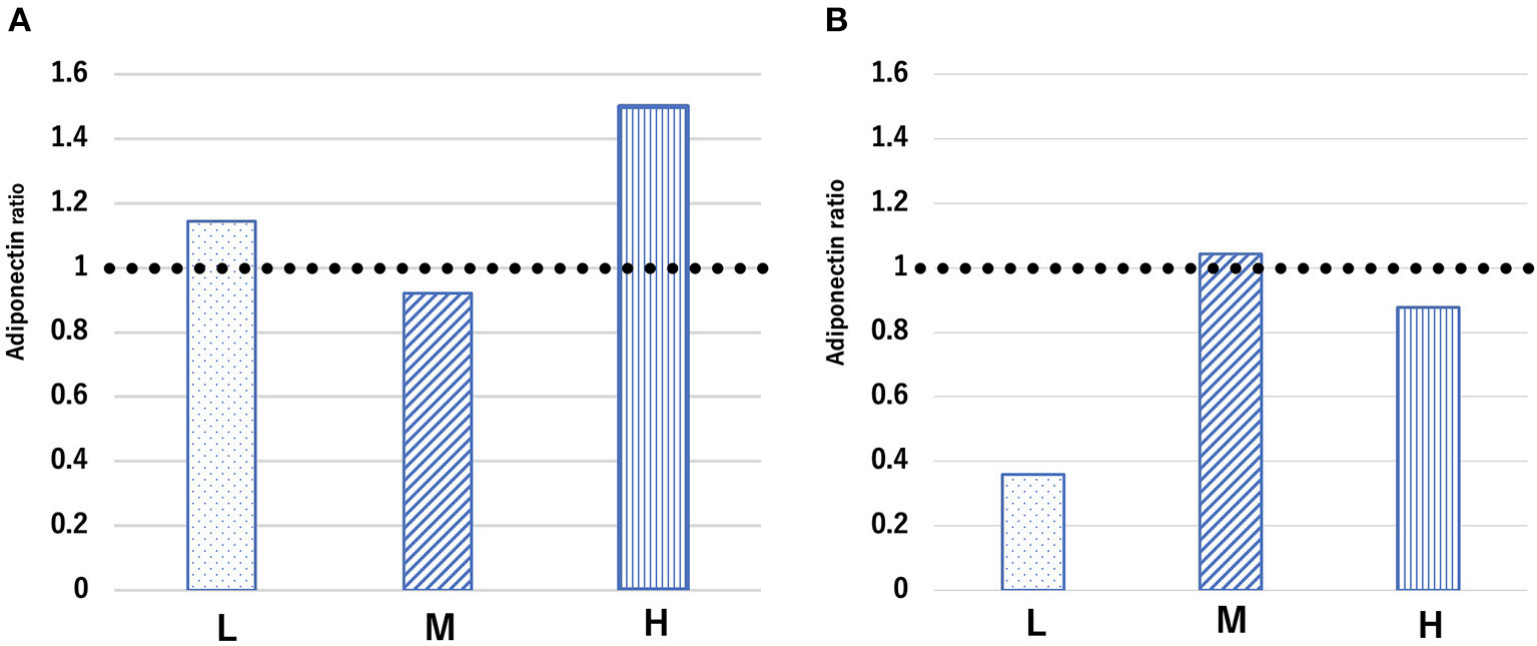
Ratio of average adiponectin concentration [ratio = adiponectin concentration **(A)** 24 h and **(B)** 48 h after cecal ligation and puncture (CLP) to adiponectin concentration before CLP] in each group. Groups L, M, and H denote low-fat, medium-fat, and high-fat diet-fed mice, respectively.

The blood concentration ratio of adiponectin is expressed as the average between groups before and after treatment rather than comparisons between individuals. The ratio of the average adiponectin concentration at 24 h after CLP to that before CLP in the L, M, and H groups was 1.1, 0.9, and 1.5, respectively (Figure 7A). Conversely, the ratio of the average concentration of adiponectin at 48 h after CLP to that before CLP was 0.35, 1.04, and 0.87 in the L, M, and H groups, respectively (Figure 7B). The concentration of adiponectin in each group of mice before and after CLP is shown in Supplementary Table 4.

### Sdc-1

**Figure 8:**
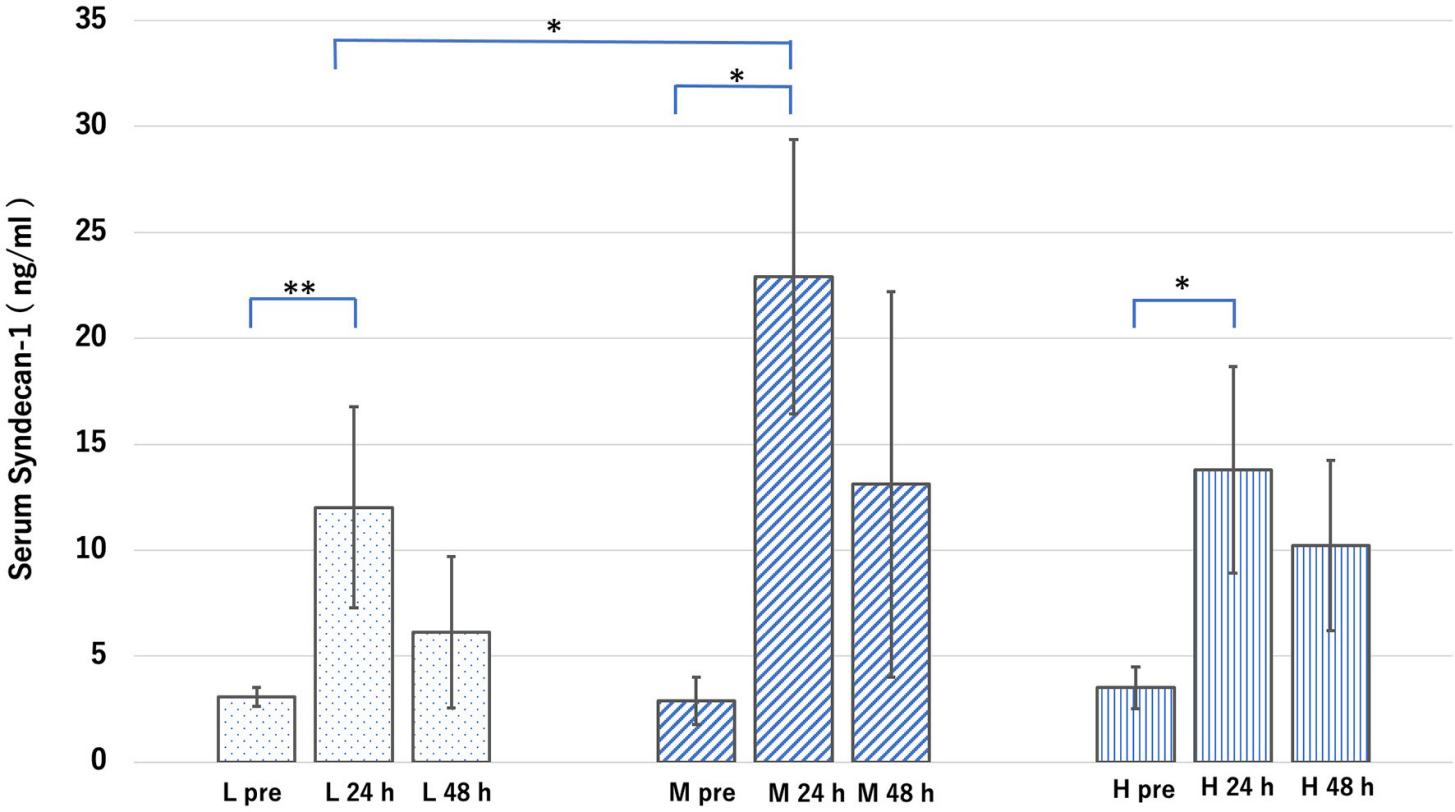
Groups L, M, and H denote low-fat, medium-fat, and high-fat diet-fed mice, respectively. L pre is the average value of the blood concentration of syndical-1 (Sdc-1) before sepsis in Group L. L 24 h is the average value of the blood concentration of Sdc-1 24 h after sepsis in Group L. L 48 h is the average value of the blood concentration of Sdc-1 48 h after sepsis in Group L. The other groups are defined in the same manner. Serum concentration of Sdc-1 in each group before and after cecal ligation and puncture. Data were analyzed using Tukey–Kramer honestly significant difference test. *p < 0.05, **p < 0.01.

Under normal conditions, the blood concentration of Sdc-1 in the L, M, and H groups was 3.0 ± 0.45, 2.8 ± 1.1, and 3.5 ± 1.0 ng/ml, respectively. Under septic conditions, the blood concentration of Sdc-1 at 24 h after CLP in the L, M, and H groups was 12.0 ± 4.7, 22.9 ± 6.4, and 13.7 ± 4.8 ng/ml, respectively, whereas that at 48 h after CLP was 6.1 ± 3.5, 13.1 ± 9.1, and 10.2 ± 4.0 ng/ml, respectively (Figure 8). Therefore, the blood concentration of Sdc-1 at 24 h after CLP was significantly higher than that before CLP in all groups. However, the blood concentration of Sdc-1 at 48 h after CLP was not significantly different from that before CLP in all groups. Furthermore, the blood concentration of Sdc-1 in the M group at 24 h after CLP was significantly higher than that in the L group at 24 h after CLP.

## Discussion

The functions of endothelial cells in obesity and metabolic syndrome differ. Metabolic syndrome is a condition in which insulin resistance leads to the development of hypertension, hyperlipidemia, atherosclerosis, and cardiovascular disease and is closely related to GCX disorders (33–35), especially in the early stages (36). A previous study indicated that blood concentrations of Sdc-1 and heparan sulfate (GCX components) are high in patients undergoing dialysis, and the relationship between atherosclerosis and GCX damage has also been reported (37). In contrast, obesity can transition into metabolic syndrome in high-risk patients; however, the normalcy of GCX in obese individuals has not been investigated. The aim of this study was to evaluate endothelial cell function and the physiological function of GCX in a simple obesity model. This study provides novel insights into the obesity paradox. Previous studies reported that the GCX layer collapses and increases vascular permeability in severe conditions, including sepsis (12, 38), massive bleeding (23), acute heart failure (39), and pneumonia (40). This can lead to interstitial edema, impaired microcirculation, and reduced organ perfusion, resulting in critical multiorgan failure (12). Uzawa et al. (22) reported that the disruption of GCX in small arteries with a vessel diameter of 20 μm altered vascular permeability in the interstitial space in the microcirculation. The disruption of GCX causes shedding of hyaluronan and Sdc-1, and circulating glycosaminoglycans enhance the existing inflammation by binding to a variety of molecules and triggering the release of inflammatory mediators, such as chemokines, from within the ESL. These may lead to increased vascular permeability and impaired microcirculation in capillaries and post-capillary vessels, resulting in various endothelial cell disorders (41). Regarding GCX thickness and vessel diameter, previous studies have shown that the GCX layer tends to thicken as the vessel diameter increases (Table 2), which was also the case for the GCXI of all groups in this study (Figure 4). The positive correlation between the GCXI and vessel diameter was stronger in the M and H groups than in the L group, suggesting that there may be a stronger tendency for the original GCX to thicken as the vessel diameter increases in the M and H groups. In this situation, GCX is impaired at a certain rate due to stress from sepsis; therefore, it is likely that the intact GCX will remain thicker in the H and M groups than in the L group. This result may explain one aspect of the pathogenesis of the obesity paradox. In the M and H groups, the greater the diameter of the vessel, the thicker the GCX compared with that in the L group, suggesting some tolerance for the phenomenon of inflammatory stress-induced GCX shedding.

In this study, we implanted the CCW in the CLP model and observed each group using an intravital fluorescent microscope; however, we could not obtain clear images because of edema resulting from inflammation. Thus, further studies are required for a better understanding of the *in vivo* GCX impairment and the obesity paradox in the pathogenesis of sepsis.

Adiponectin has GCX relevance and exerts anti-inflammatory and beneficial effects on vascular barrier function after trauma (19, 42). Some reports have indicated a relationship between adiponectin concentration in the blood and the prognosis of critical illnesses (e.g., the poor prognosis of burns and pancreatitis is related to a decrease in adiponectin concentration) (43, 44). However, another report suggested that adiponectin concentration is not correlated with inflammatory markers (45). Additionally, a poor prognosis in severe diseases may be related to elevated adiponectin concentrations (46–49). Thus, it remains unclear whether blood adiponectin concentrations affect the severity or prognosis of critical conditions. At the very least, adiponectin seems to play a role as a physiological defense mechanism in sepsis (50). In the present study, CLP-induced septic conditions promoted elevations in the adiponectin ratio in the L and H groups after 24 h of CLP, which then decreased after 48 h, whereas the adiponectin ratio in the M group decreased slightly after 24 h of CLP and increased slightly after 48 h. The adiponectin ratio in the M group was stable at both 24 and 48 h. Moreover, the adiponectin concentration at 48 h was significantly lower than that before CLP in only the L group (Supplementary Table 4).

The mechanism of cancer metastasis due to GCX dysfunction was recently been reported (51). Furthermore, another study reported the relationship between various cancers, including hepatocellular carcinoma (52), cervical carcinoma (53), colorectal carcinoma (54), and breast carcinoma (55), and blood levels of adiponectin (53). Additionally, blood levels of adiponectin are reportedly related to cancer severity (56). Adiponectin is generally considered to function in a suppressive manner against cancer cells, and it is speculated that there is a relationship between the blood level of adiponectin and GCX. It is possible that a high-fat diet causes adipocytes to swell and hypersensitize adiponectin secretion, thereby enhancing the induced anti-inflammatory mechanism and acting protectively against GCX.

The blood concentration of Sdc-1 at 24 h after CLP was significantly higher in the M group than in the L group, making it reasonable to assume that the collapse of the GCX in the M group was greater than that in the L group. After 48 h of CLP, the Sdc-1 concentration did not differ significantly between groups; however, the ratio of the blood concentration of adiponectin at 24 and 48 h was the most stable in the M group. The reason for this contradictory phenomenon might be the directly proportional relationship between the vessel diameter and the intact GCX thickness. Because the GCXI under normal conditions was significantly thicker in groups M and H than in group L, it is possible that even if GCX disruption was more in group M than in group L, there would be less damage to the actual endothelial cells in groups M and H. However, it was difficult to demonstrate this mechanism in this study, because we were unable to stain GCX and measure its index in a CLP-induced sepsis model.

Further studies using advanced image-analysis techniques, such as observation confocal microscopy, are needed to elucidate the relationship between adiponectin concentration and GCX thickness. Maintaining constant secretion of adiponectin, which has anti-inflammatory effects, may be advantageous in conditions, such as sepsis, where inflammation leads to increased vascular permeability. This result might explain another aspect of the obesity paradox. In most clinical cases, the prognosis in severe critical conditions may actually be worse due to peripheral circulatory failure and high insulin resistance caused by the large number of adipocytes.

However, the present study has certain limitations. First, the obesity model itself represents a limitation, as there is currently no mouse-related index of obesity similar to the human BMI (e.g., moderate obesity equals a BMI of 27–30). Therefore, it remains unclear where the H and M groups correspond to the human BMI scale or if an exact obesity paradox model can be produced. The blood pressure of mice in groups M and group H was statistically significantly higher than that in group L. The mean blood pressure in groups M and H was approximately 1.1 times higher than that in group L (Table 1). Although hypertension and atherosclerosis have been reported to be associated with GCX disruption (57), it is not clear from our experiment to what degree hypertension affected GCX, and the effect of blood pressure as well as BMI on GCX in humans may be different in our study model. The second limitation is the CCW in sepsis. Through the CCW window, we were unable to observe the vessel wall in the M and H groups, because there were too many fat cells. Although the vessel wall in pre-CLP conditions could be observed in the CCWs, the CCWs in the sepsis model exhibited strong edema; therefore, clear images could not be obtained, and the GCXI according to vessel diameter could not be measured. Therefore, it was not possible to observe the *in vivo* effect of GCX on direct sepsis injury. Using a multi-photon laser scanning microscope to measure the GCXI in the obese group with sepsis might allow elucidation of the *in vivo* effects of adiponectin on GCX. However, because this equipment is expensive and given the concerns of photo-damage to fragile brain tissue, we used an ordinary fluorescence light microscope. Third, the accuracy of the GCX measurement method was a limitation, as it is difficult to observe and analyze GCX *in vivo*. Although GCX thickness has been measured in various ways in previous studies, different methods of measurement and correcting measurement errors have been devised (Supplementary Table 5). The indirect method of GCX measurement using fluorescent labeling of polymer dextran and staining the vessel lumen may overestimate or underestimate the errors due to auto-vasomotion. Moreover, measurements using the extent of red blood cell migration as a proxy for the lumen of blood vessels might cause similar problems. Therefore, we performed GCX measurement using WGA-FITC in this study, as it directly fluorescently labels GCX, which eliminates the effect of auto-vasomotion. However, this may cause inaccuracy in the positioning of the GCX edge. Because the fluorescence-intensity signal from FITC-WGA included all fluorescent signals on and in a curved luminal vessel surface area, an accurate GCX thickness could not be obtained; however, this signal might be an indicator of GCX presence according to the accumulation of GCX components, such as glycosaminoglycans. In this study, we used the GCXI as a measure of GCX size and used this value for a relative comparison. Furthermore, measurements of the GCXI showed a similar relationship between GCX thickness and vessel diameter in previous studies (Table 2, Figure 4D), where GCX measurements increased with vessel diameter. The GCXI is calculated based on a simple measurement of the fluorescence intensity of the total edge of the blood vessel, which is beyond the limit of the resolution of optical microscopy and may not precisely represent the GCX thickness. Considering that the major variable in the size of the GCXI is the GCX thickness, we assumed that the GCXI has a close relationship to GCX thickness.

In conclusion, we found that the GCXI of obese groups in the non-sepsis model was thicker than that of the low-fat group for all vessel diameters, and that the positive correlation was also stronger. Additionally, the circulating blood concentration of adiponectin was stable in the medium-fat group, although Sdc-1 at 24 h after CLP was high. These findings suggest that the thickness of the original GCX might play an important role in the obesity paradox by having a beneficial effect on pathological changes in critical conditions.

## Data Availability Statement

The original contributions presented in the study are included in the article/Supplementary Material, further inquiries can be directed to the corresponding author/s.

## Ethics Statement

The animal study was reviewed and approved by Committee for Animal Experiments at the National Institute of Public Health (Protocol Number 31-002).

## Author Contributions

SM conceived and designed this study, secured competitive funding, wrote the manuscript, and conducted all animal studies under the guidance of AU. KU contributed to the analysis of all data and writing of the manuscript. MS, TA, and TY performed the experiments and created the figures. HM and TY proposed the research draft and developed an experimental plan for the entire study. AU performed the experiments, data analysis, and image processing and analysis. All authors contributed to the article and approved the submitted version.

## Funding

This work was supported by a Grant-in-Aid for Young Scientists (B) (Grant Number JP16K15680) and a Grant-in-Aid for Exploratory Research (Grant Number JP19K18251).

## Conflict of Interest

The authors declare that the research was conducted in the absence of any commercial or financial relationships that could be construed as a potential conflict of interest.

## Publisher's Note

All claims expressed in this article are solely those of the authors and do not necessarily represent those of their affiliated organizations, or those of the publisher, the editors and the reviewers. Any product that may be evaluated in this article, or claim that may be made by its manufacturer, is not guaranteed or endorsed by the publisher.
