# Supplementary material for: Vascular Endothelial Glycocalyx Plays a Role in the Obesity Paradox According to Intravital Observation
Source: Front Cardiovasc Med. 2021 Nov 2;8:727888. doi: 10.3389/fcvm.2021.727888 (PMC8593246; doi:10.3389/fcvm.2021.727888)
Supplement: Supplementary file 1 [file Data_Sheet_1.docx]

Supplementary Material

#
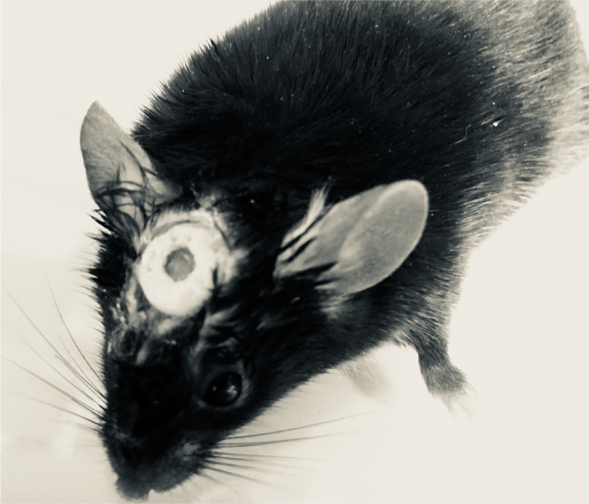
Supplementary Figures

**Supplementary Figure 1. Mouse with chronic cranial window.**

We observed cerebral micro-vessels by CCW. A 3.5-mm hole was drilled in the skull of a mouse, and a glass slide was placed on top of the CCW and fixed with resin. A 5-mm diameter cover glass was attached to allow observation of cerebral blood vessels. CCW enables long-term observation of cerebral microcirculation *in vivo*.


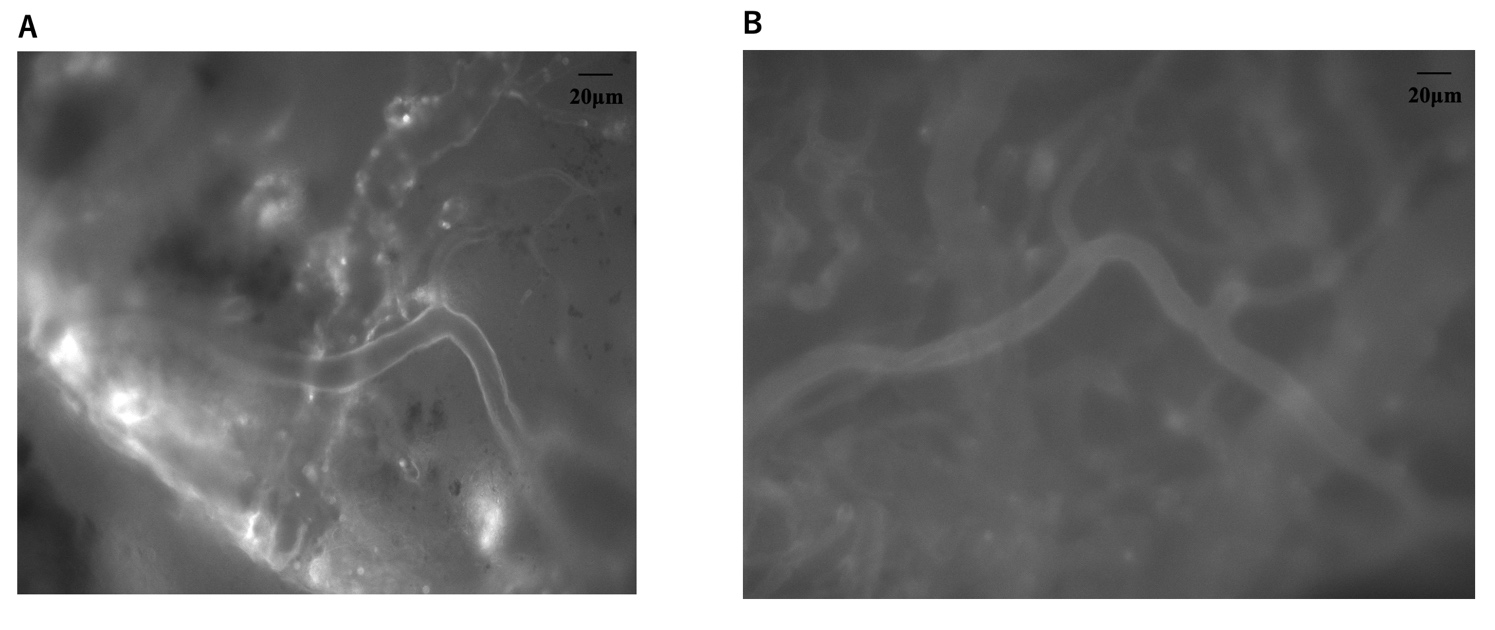


**Supplementary Figure 2. Images of the cerebral endothelial GCX under normal and septic conditions.**

We illuminated the cerebral endothelial GCX using the fluorescein isothiocyanate-labeled wheat germ agglutinin (FITC-WGA) lectin *in vivo* and observed them through a cranial window using an intravital microscope. Cerebral endothelial GCX layers under (**A**) normal and (**B**) septic conditions. Clear images were difficult to obtain under septic conditions. These two images show the same blood vessel of the same mouse before and after CLP.

**
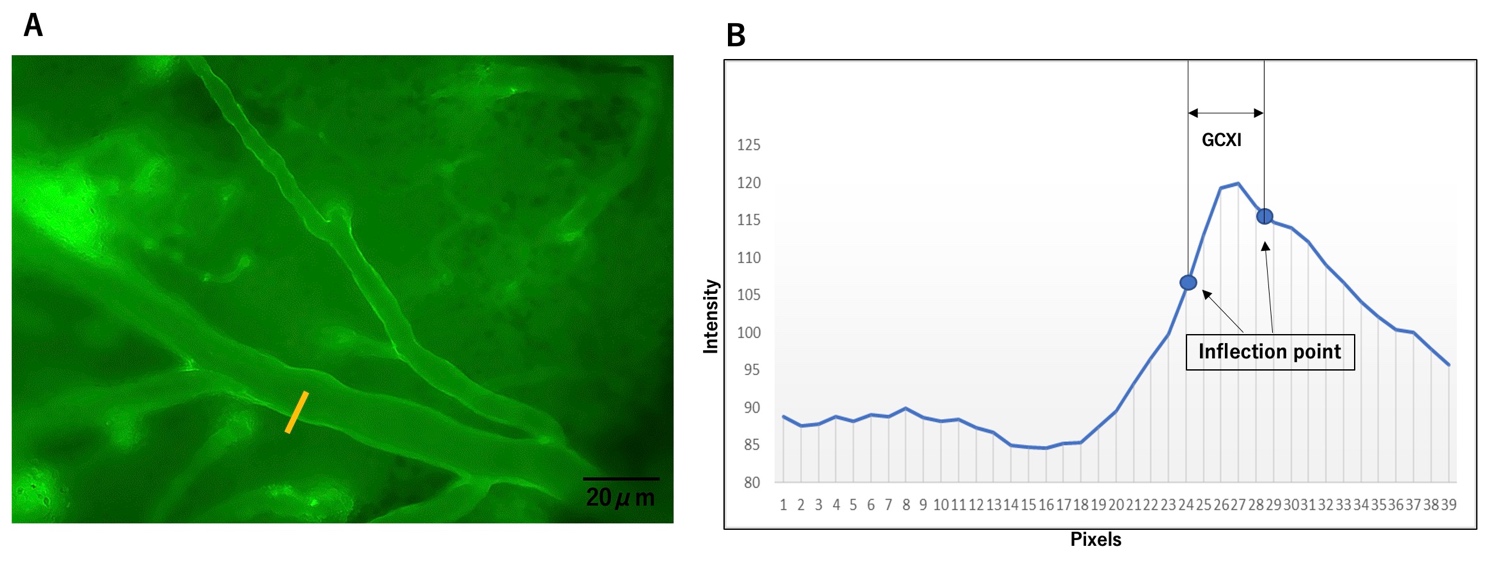
**

**Supplementary Figure 3. Measurement of the GCXI.**

We illuminated the endothelial GCX layer using FITC. (**A**) A line perpendicular to the vessel wall was drawn and its fluorescence intensity measured. The same procedure was performed three times with different lines. The average intensity profile of the measurement was fitted with a Gaussian curve, and the inflection point was calculated based on the fitted curve. (**B**) The two inflection points were subtracted from each other, and the value was considered to represent FITC-WGA lectin accumulation in the endothelial GCX layer. We defined the size of the FITC-WGA-positive layer as the GCXI, which represents the accumulation of several GCX components. One pixel is equivalent to 0.3774 μm.

# Supplementary Tables

**Supplementary Table 1.** Age (weeks)/weight data of all mice.

Data of all mice used in this study [body fat percentage, blood biochemical tests, GCXI, Sdc-1 and adiponectin measurements, and 48-h mortality). Groups L, M, and H denote low-fat mice, medium-fat mice, and high-fat mice, respectively. The data include the number of weeks and body weight of all mice used in these experiments and were analyzed using the Tukey–Kramer honestly significant difference test. N.S., not significant.

**Supplementary Table 2.** Blood biochemical tests for each group of mice.

|  | WBC | HGB | PLT | ALB | CRE | GLU | T-CHOL | HDL | TRIG | LDL |
| --- | --- | --- | --- | --- | --- | --- | --- | --- | --- | --- |
| L (N = 7) | 2.83 ± 1.25 | 13.9 ± 0.9 | 583 ± 168 | 3.24 ± 0.53 | 0.285 ± 0.12 | 340 ± 94.7 | 96.1 ± 35.7 | 67.9 ± 29 | 92.3 ± 33.8 | 9.86 ± 7.99 |
| M (N = 7) | 2.96 ± 0.88 | 13.5 ± 0.99 | 654 ± 153 | 3.3 ± 0.73 | 0.3 ± 0.12 | 402 ± 94.6 | 162 ± 31.4** | 90 ± 6.08 | 131 ± 91.5 | 25 ± 9.64 |
| H (N = 10) | 3.13 ± 3.07 | 13.6 ± 1.01 | 607 ± 124 | 3.2 ± 0.55 | 0.3 ± 0.18 | 356 ± 77.6 | 144 ± 24.2* | 88.6 ± 15.6 | 136 ± 64.6 | 26 ± 11.7* |

Groups L, M, and H denote low-fat mice, medium-fat mice, and high-fat mice, respectively. *p < 0.05, **p < 0.01, Tukey–Kramer honestly significant difference test.

WBC, white blood cell; HGB, hemoglobin; PLT, platelet; ALB, albumin; CRE, creatinine; GLU, glucose; T-CHOL, total cholesterol; HDL, high-density lipoprotein; TRIG, triglyceride; LDL, low-density lipoprotein.

**Supplementary Table 3.** Body weight of mice in each group before and after CLP.

|  | Before CLP | 24 h After CLP | 48 h After CLP |
| --- | --- | --- | --- |
| L (N=11) | 23.7 ± 0.79g | 21.1 ± 0.70g | 20.4 ± 1.00g |
| M(N=10) | 25.13 ± 1.94g | 23.1 ± 2.08g | 22.2 ± 1.90g |
| H (N=9) | 28.1 ± 2.58g | 26.0 ± 2.28g | 25.0 ± 2.25g |

Groups L, M, and H denote low-fat mice, medium-fat mice, and high-fat mice, respectively. Data represent the mean ± standard error.

**Supplementary Table 4.** Adiponectin concentration in each group of mice before and after CLP.

A

| Group | Before CLP | 24 h After CLP |
| --- | --- | --- |
| L | 5.19 ± 0.39μg/ml (n=5) | 5.94 ± 1.65μg/ml (n=5) |
| M | 4.69 ± 0.62μg/ml (n=4) | 4.33 ± 1.30μg/ml (n=4) |
| H | 4.16 ± 1.48μg/ml (n=5) | 6.26 ± 1.76μg/ml (n=5) |

B

| Group | Before CLP | 48 h After CLP |
| --- | --- | --- |
| L | 1.66 ± 0.24μg/ml (n=5) | 0.59 ± 0.25**μg/ml (n=5) |
| M | 0.99 ± 0.15μg/ml (n=2) | 1.03 ± 0.56μg/ml (n=6) |
| H | 0.96 ± 0.59μg/ml (n=3) | 0.84 ± 0.41μg/ml (n=5) |

Adiponectin concentrations determined before and 24 h (A) and 48 h (B) after CLP. Groups L, M, and H denote low-fat mice, medium-fat mice, and high-fat mice, respectively. Data represent the mean ± standard deviation. **p < 0.01 vs. before CLP. Adiponectin concentration in the L group at 48 h after CLP was significantly lower than that in the L group before CLP.

**Supplementary Table 5.** Evaluation of GCX thickness based on previous study values.

| **Year** | **Author** | **Method used to measure GCX thickness** | **Method used to correct errors in GCX thickness measurement** |
| --- | --- | --- | --- |
| 1996 | Vink | Dye-exclusion method | Correct caliper bar thickness |
| 2009 | Potter | Microviscometric analysis | Not described |
| 2011 | Eno E Ebong | Electron Microscopy | Not described |
| 2013 | Lipowsky | Dye-exclusion method,  Image analysis method (inflection point) | Not described |
| 2012 | Wan-Y Yen | Image analysis method (half- maximum) | Not described |
| 2013 | Ivo Torres Filho | Dye-exclusion method,  Image analysis method (inflection point) | Not described |
| 2013 | Michele D. Savery | Microviscometric analysis | Not described |
| 2016 | Ivo P. Torres Filho | Dye-exclusion method,  Image analysis method (inflection point) | Not described |
| 2017 | Jin-Hui | Image analysis method (inflection point) | Three iterations to reduce tracing error |
| 2017 | Jin-Hui | Image analysis method (inflection point) | Three iterations to reduce tracing error |
| 2017 | Jin-Hui | Image analysis method (inflection point) | Three iterations to reduce tracing error |
| 2017 | Kataoka | Image analysis method (half- maximum) | Not described |
| 2018 | Xiaoyuan | Dye-exclusion method | Not described |

Evaluations are based on descriptions provided in Fig. 4D. The dye-exclusion method measures the diameter of anatomical capillaries using digital calipers in bright-field biomicroscopy images and compares it with the value of the functional diameter of capillaries obtained from measurement of the width of the capillary lumen occupied by FITC-dextran according to fluorescence microscopy. Microviscometric analysis is used to estimate GCX thickness by hydrodynamic analysis of micro-particle image velocimetry data. The image-analysis method (inflection point) estimates GCX thickness by defining the position of the inflection point of the dye intensity curve with respect to the vertical line of fluorescently stained endothelial cells as the outer edge of the GCX layer. The image-analysis method (half- maximum) is used to estimate GCX thickness by defining thickness as the distance between two points, where the fluorescence intensity of the dye intensity curve for the vertical line of fluorescently stained endothelial cells decreases by 50%.
